# Comparison of the efficacy and safety of repeated hepatectomy and radiofrequency ablation in the treatment of primary recurrent liver cancer: a meta-analysis

**DOI:** 10.1186/s12957-022-02649-4

**Published:** 2022-06-06

**Authors:** Zhichao Chen, Jiefang Wang, Yonghua Lin

**Affiliations:** 1 Department of Hepatobiliary and Pancreatic Surgery, the Second Affiliated Hospital of Fujian Medical University, Quanzhou, 362000 China; 2 Department of Radiology, Quanzhou First Hospital Affiliated to Fujian Medical University, Quanzhou, 362000 China

**Keywords:** Repeated hepatectomy, Radiofrequency ablation, Recurrent liver cancer, Meta-analysis

## Abstract

**Background:**

Since there is still controversy about the comparison of the efficacy and safety of RH and RFA in the treatment of recurrent liver cancer, we conducted a meta-analysis to compare the efficacy and safety, in order to provide evidence-based evidence for future research and clinical treatment.

**Methods:**

We searched PubMed, Embase, and Cochrane Library from the establishment of the database to Feb 2021. We included studies that reported liver cancer patients underwent repeated hepatectomy (RH) or radiofrequency ablation (RFA), and we excluded duplicate publications, research without full text, incomplete information, or inability to conduct data extraction, animal experiments, reviews, and systematic reviews. The STATA 15.1 was used to analyze the data.

**Results:**

The pooled results show that the 3-year and 5-year overall survival (OS) rate of the repeated hepatectomy group was significantly higher than the radiofrequency ablation group (odds ratio (OR) = 1.95, 95% confidence interval (CI):1.47–2.60, *P* ≤ 0.001; *OR* = 1.65, 95% *CI*: 1.12–2.43, *P* = 0.012). Similarly, the pooled results show that the 3-year and 5-year disease-free survival (DFS) rate of the repeated hepatectomy group was significantly higher than the radiofrequency ablation group (*OR* = 1.73, 95% *CI*: 1.30–2.31, *P* ≤ 0.001; *OR* = 1.84, 95% *CI*: 1.38–2.49, *P* ≤ 0.001). However, there is no significant difference in the 1-year OS and DFS rate of repeated hepatectomy group and radiofrequency ablation group. Additionally, the pooled results show that the postoperative Clavien-Dindo (CD) grade II or higher complication rate of the repeated hepatectomy group was significantly higher than the radiofrequency ablation group (*OR* = 2.80, 95% *CI*: 1.37–5.75, *P* = 0.005).

**Conclusion:**

Based on the pooled results of 8 existing retrospective studies, RH has a higher OS rate and DFS rate in the treatment of recurrent liver cancer, while the postoperative complication rate of RFA is lower. When survival is the primary goal, RH should be the first choice for recurrent liver cancer.

**Supplementary Information:**

The online version contains supplementary material available at 10.1186/s12957-022-02649-4.

## Introduction

Primary liver cancer is the fourth most common malignant tumor and the second leading cause of tumor death in my country [1]. Treatment options for liver cancer include intended preoperative TAE [2], TAE combined with portal vein embolization [3], and hepatectomy. Hepatectomy is currently the most important method for curative treatment of primary liver cancer [4]. However, the 5-year recurrence rate after hepatectomy for primary liver cancer is greater than 70%, so the curative effect is not ideal [5]. It is reported that repeated hepatectomy (RH) can be performed safely and is associated with long-term survival in a subset of patients with recurrent liver cancer. Increasing studies have shown that RH is currently the primary treatment for recurrent liver cancer [6, 7]. Additionally, radiofrequency ablation (RFA) has gradually become another treatment for recurrent liver cancer due to its characteristics of small trauma and quick recovery [8]. The results of a meta-analysis by Jin et al. showed that laparoscopic hepatectomy is preferred over RFA treatment with a better radical effect, but RFA treatment is more beneficial with smaller trauma, development of less complications, and shorter operating time [9]. Wei et al. [10] found that both repeat hepatectomy and RFA are shown to be effective and safe for the treatment of recurrent hepatocellular carcinoma located in the subcapsular region. However, the clinical treatment of recurrent liver cancer has not yet reached a consensus, and there is still controversy about the comparison of the efficacy and safety of RH and RFA in the treatment of recurrent liver cancer. Therefore, this study conducted a meta-analysis by systematically reviewing relevant literature to compare the efficacy of RH and RFA in the treatment of recurrent liver cancer, in order to provide evidence-based evidence for future research and clinical treatment.

## Methods

### Protocol registration

This protocol has been registered, the registration number is INPLASY202250119, and the DOI number is 10.37766/inplasy2022.5.0119.

### Literature inclusion and exclusion criteria

#### Inclusion criteria

- Study object: Patients with recurrent liver cancer
- Intervention measures: The observation group underwent repeated hepatectomy.
- Control: The control group underwent radiofrequency ablation.
- Outcome indicators: The 1-, 3-, and 5-year overall survival (OS) rates and disease-free survival (DFS) rates
- Study design: Randomized controlled trials or cohort studies or case-control studies, the language is limited to English.

#### Exclusion criteria

- Primary liver cancer or other nonrecurrent liver cancer
- The observation group did not receive repeated hepatectomy, or the control group did not receive radiofrequency ablation.
- Rate values for OS or DFS were not reported or could not be extracted from the study; duplicate publication, research without full text, incomplete information, or inability to conduct data extraction, animal experiments, reviews, and systematic reviews.

### Search strategy

In this meta-analysis, we searched PubMed, Embase, and Cochrane Library from establishment of the database to Feb 2021. The search terms are mainly as follows: ('liver neoplasms' OR 'liver neoplasm' OR 'hepatic neoplasms' OR 'hepatic neoplasm' OR 'cancer of liver' OR 'hepatocellular cancer' OR 'hepatocellular cancers' OR 'hepatic cancer') AND (recurrent OR recurrence OR relapse OR recurring) AND ('radiofrequency ablation' OR 'radio frequency ablation' OR 'radio-frequency ablation') AND ('repeated hepatic resection' OR 're-hepatectomy' OR 'repeat hepatectomy' OR 'hepatic resection' OR 'repeated resection' OR 're-resection' OR 'liver resection' OR 'surgical resection' OR 'redo hepatectomy' OR 'repeat liver resection'). The detail search strategy has been shown in Additional file 2.

### Literature screening and data extraction

The literature search, screening (title and abstract screening and full text screening), and information extraction were all independently completed by two researchers. When there were doubts or disagreements, the decision was made after discussion with a third person. Extracted data included the author, year, study area, research type, number of cases, and the indicators for evaluating outcome, including 1-year OS rate, 3-year OS rate, 5-year OS rate, 1-year DFS rate, 3-year DFS rate, 5-year DFS rate, and postoperative Clavien-Dindo (CD) grade II or higher complication rate.

### Literature quality assessment

Two researchers independently conducted literature quality evaluations using the Newcastle-Ottawa Scale (NOS) for cohort study [11]. NOS includes 4 items (4 points) for “Research Subject Selection,” 1 item (2 points) for “Comparability between Groups,” and 3 items (3 points) for “Result Measurement,” with a full score of 9 points, and ≥ 7 is regarded as high-quality literature; < 7 is divided into lower-quality literature. When the opinions are inconsistent, it is decided through discussion or consultation with the third person. The meta-analysis was performed based on the related items of the Preferred Reporting Items for Systematic reviews and Meta-Analysis statement (PRISMA statement) [12].

### Data synthesis and statistical analysis

The STATA 15.1 [9] was used to analyze the data. OR (95% CI) was used to evaluate the difference in OS rate, DFS rate, and complication rate between RH and RFA. *I*^2^ is used to evaluate heterogeneity. If the heterogeneity test is *P* ≥ 0.1 and *I*^2^ ≤ 50%, it indicates that there is homogeneity between studies, and the fixed effects model is used for combined analysis; if *P* < 0.1, *I*^2^ > 50%, it indicates that the study, if there is heterogeneity, use sensitivity analysis to find the source of heterogeneity. If the heterogeneity is still large, use the random effects model or give up the combination of results and use descriptive analysis [10]. Funnel plot and Egger’s test were used to analyze publication bias [10].

## Results

### The results of literature search

**Fig. 1:**
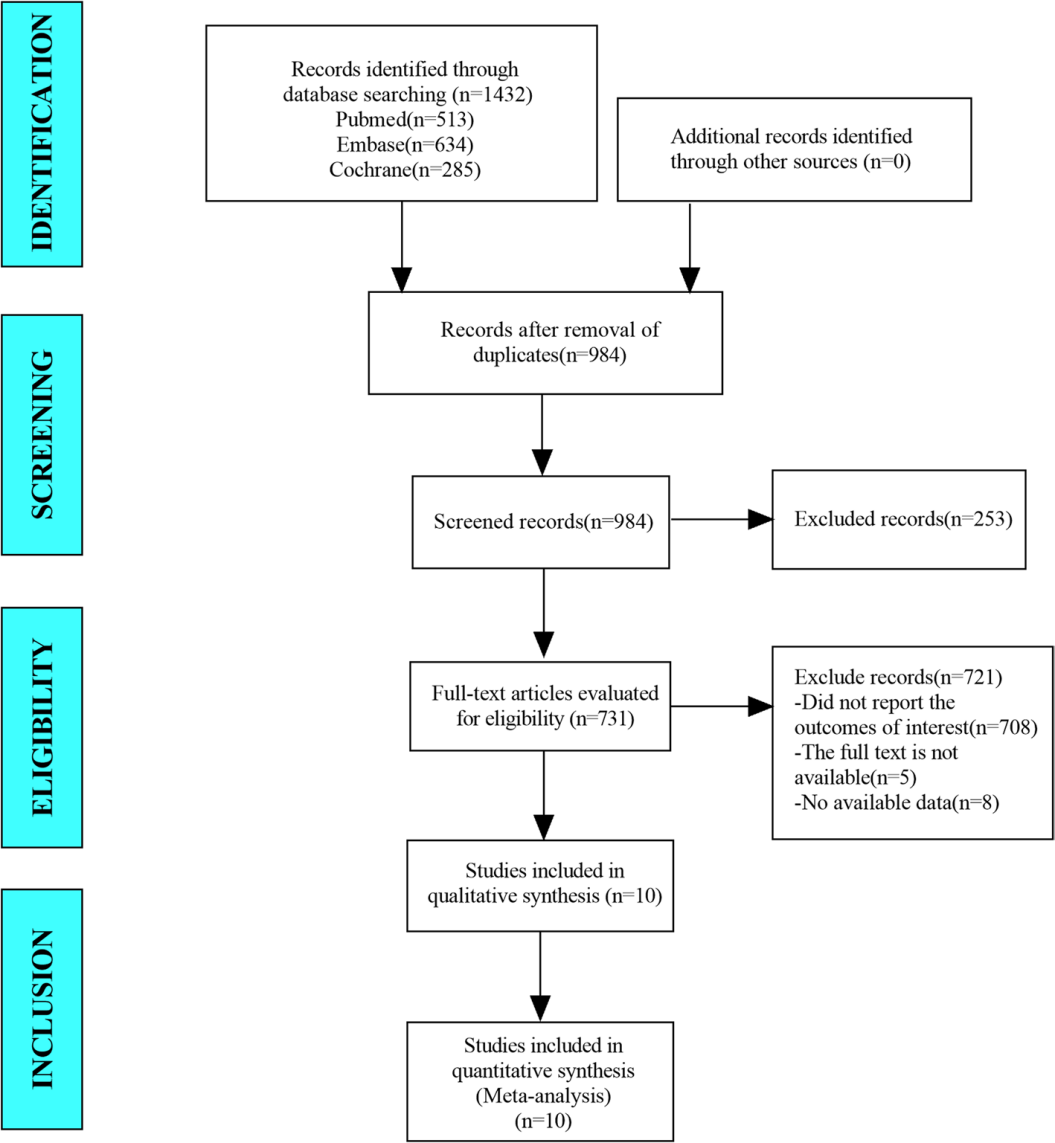
Flow diagram for selection of studies

In this study, a total of 1432 studies were retrieved from the database. After eliminating duplicate studies, 984 were obtained. After browsing titles and abstracts, 731 studies were obtained. Finally, 10 articles that can be used for meta-analysis were obtained through full-text screening (Fig. 1).

### Baseline characteristics and quality assessment of the included studies

A total of 10 cohort studies were included in this meta-analysis. The sample size of patients ranged from 26 to 290, and totally 1332 patients, including 604 patients in the repeated hepatectomy group and 728 patients in the radiofrequency ablation group. Patients in 7 studies were from China, and patients in the other two studies are from Japan and South Korea, while the patients with only one study were from Europe. The NOS score used for quality assessment is all above 7 and meets the requirements (Table 1).

**Table 1:** The baseline characteristics quality assessment of the included studies

Author	Year	Research type	Study area	Number of patients		Gender (male/female)		Age (year)		NOS score
				Repeated hepatectomy	Radiofrequency ablation	Repeated hepatectomy	Radiofrequency ablation	Repeated hepatectomy	Radiofrequency ablation	
Umeda et al. [13]	2010	Cohort	Japan	29	58	/	/	/	/	8
Ho et al. [14]	2012	Cohort	China	54	50	40/14	39/11	56.3 ± 12.3	61.0 ± 11.1	7
Chan et al. [15]	2012	Cohort	China	29	45	/	/	52.0 (38.0–79.0)	59.0 (36.0–80.0)	7
Eisele et al. [16]	2013	Cohort	Germany	27	27	15/12	20/7	60.0 ± 17.0	68.0 ± 7.0	7
Huang et al. [17]	2015	Cohort	China	15	11	9/6	8/3	/	/	8
Song et al. [18]	2015	Cohort	Korea	39	78	31/8	58/20	52.5 ± 9.8	53.6 ± 10.9	7
Wang et al. [19]	2015	Cohort	China	128	162	113/15	148/14	49.2 ± 10.1	51.9 ± 10.9	7
Sun et al. [20]	2017	Cohort	China	43	57	34/9	38/19	60.0 (35.0–76.0)	63.0 (27.0–81.0)	8
Xia et al. [21]	2019	Cohort	China	120	120	107/13	109/11	50.0 (24.0–58.0)	52.0 (25.0–59.0)	7
Lu et al. [22]	2020	Cohort	China	120	120	108/12	104/16	50.3 ± 10.5	50.9 ± 11.6	7

### Results of meta-analysis

**Fig. 2:**
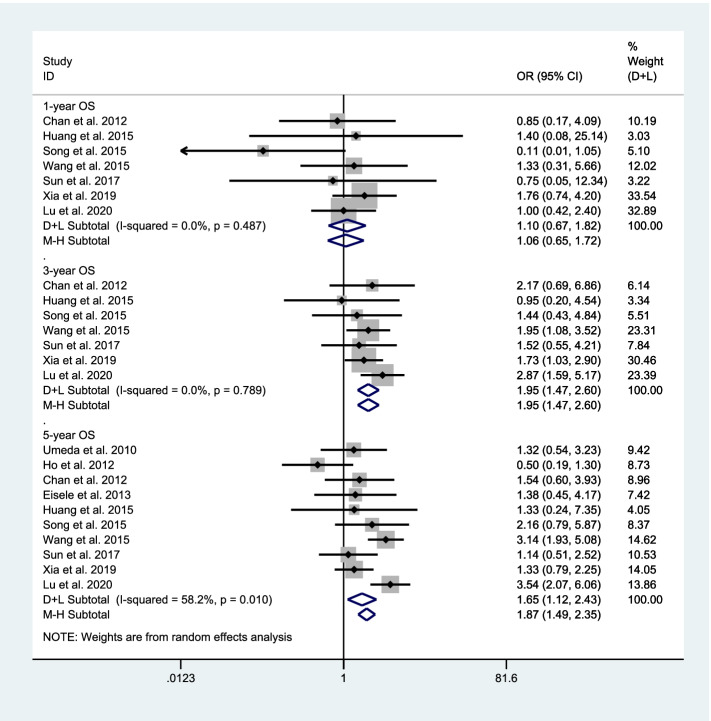
Comparison in OS rate between repeated hepatectomy and radiofrequency ablation in the treatment of recurrent liver cancer

We first explored the difference in OS rate between RH and RFA. There are 7 studies [15, 17–22], including 1087 patients, compared the 1-year OS rate between RH and RFA. Since there is no significant heterogeneity (*I*^2^ = 0.0%, *P* = 0.487 > 0.1), a meta-analysis was conducted through a fixed effects model. The pooled results show that there was no significant difference in the 1-year OS rate between RH and RFA (*OR* = 1.06, 95% CI: 0.65–1.72, *P* = 0.706) (Fig. 2). There are 7 studies [15, 17–22], including 1087 patients, compared the 3-year OS rate between RH and RFA. Since there is no significant heterogeneity (*I*^2^ = 0.0%, *P* = 0.789 > 0.1), a meta-analysis was conducted through a fixed effects model. The pooled results show that the 3-year OS rate of RH was significantly higher than RFA (*OR* = 1.95, 95% *CI*: 1.47–2.60, *P* ≤ 0.001) (Fig. 2). There are 10 studies [13–22], including 1332 patients, compared the 5-year OS rate between RH and RFA. Since there is significant heterogeneity (*I*^2^ = 58.2%, *P* = 0.010 < 0.1), a meta-analysis was conducted through a random effects model. The pooled results show that the 5-year OS rate of RH was significantly higher than RFA (*O*R = 1.65, 95% *CI*: 1.12–2.43, *P* = 0.012) (Fig. 2).

**Fig. 3:**
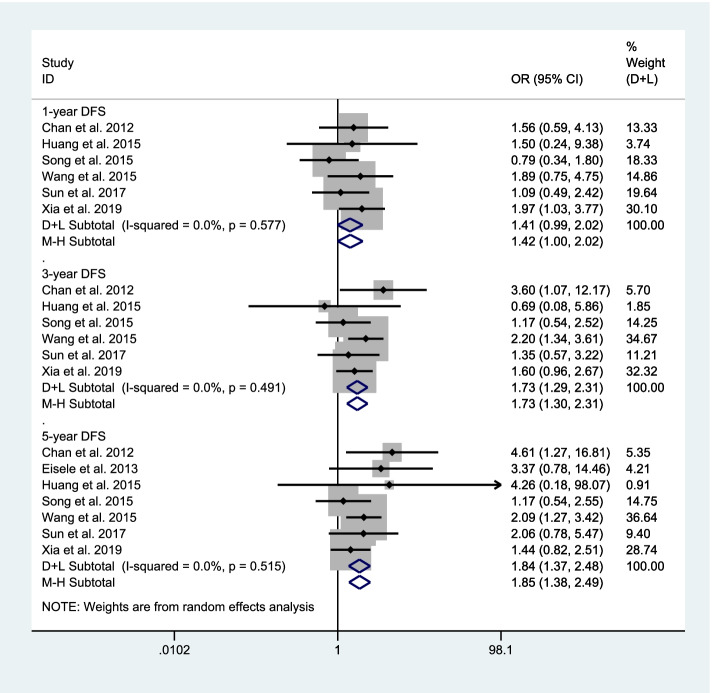
Comparison in DFS rate between repeated hepatectomy and radiofrequency ablation in the treatment of recurrent liver cancer

We continue to explore the difference in DFS rate between RH and RFA. There are 6 studies [15, 17–21], including 847 patients, compared the 1-year DFS rate between RH and RFA. Since there is no significant heterogeneity (*I*^2^ = 0.0%, *P* = 0.577 > 0.1), a meta-analysis was conducted through a fixed effects model. The pooled results show that there was no significant difference between repeated hepatectomy group and radiofrequency ablation group (*OR* = 1.41, 95% *CI*: 0.99–2.02, *P* = 0.056) (Fig. 3). There are 6 studies [15, 17–21], including 847 patients, compared the 3-year DFS rate between RH and RFA. Since there is no significant heterogeneity (*I*^2^ = 0.0%, *P* = 0.491 > 0.1), a meta-analysis was conducted through a fixed effects model. The pooled results show that the 3-year DFS rate of RH was significantly higher than RFA (*OR* = 1.73, 95% *CI*: 1.30–2.31, *P* ≤ 0.001) (Fig. 3). There are 7 studies [15–21], including 901 patients, compared the 1-year DFS rate between RH and RFA. Since there is no significant heterogeneity (*I*^2^ = 0.0%, *P* = 0.515 > 0.1), a meta-analysis was conducted through a fixed effects model. The pooled results show that the 5-year DFS rate of RH was significantly higher than RFA (*OR* = 1.84, 95% *CI*: 1.38–2.49, *P* ≤ 0.001) (Fig. 3).

**Fig. 4:**
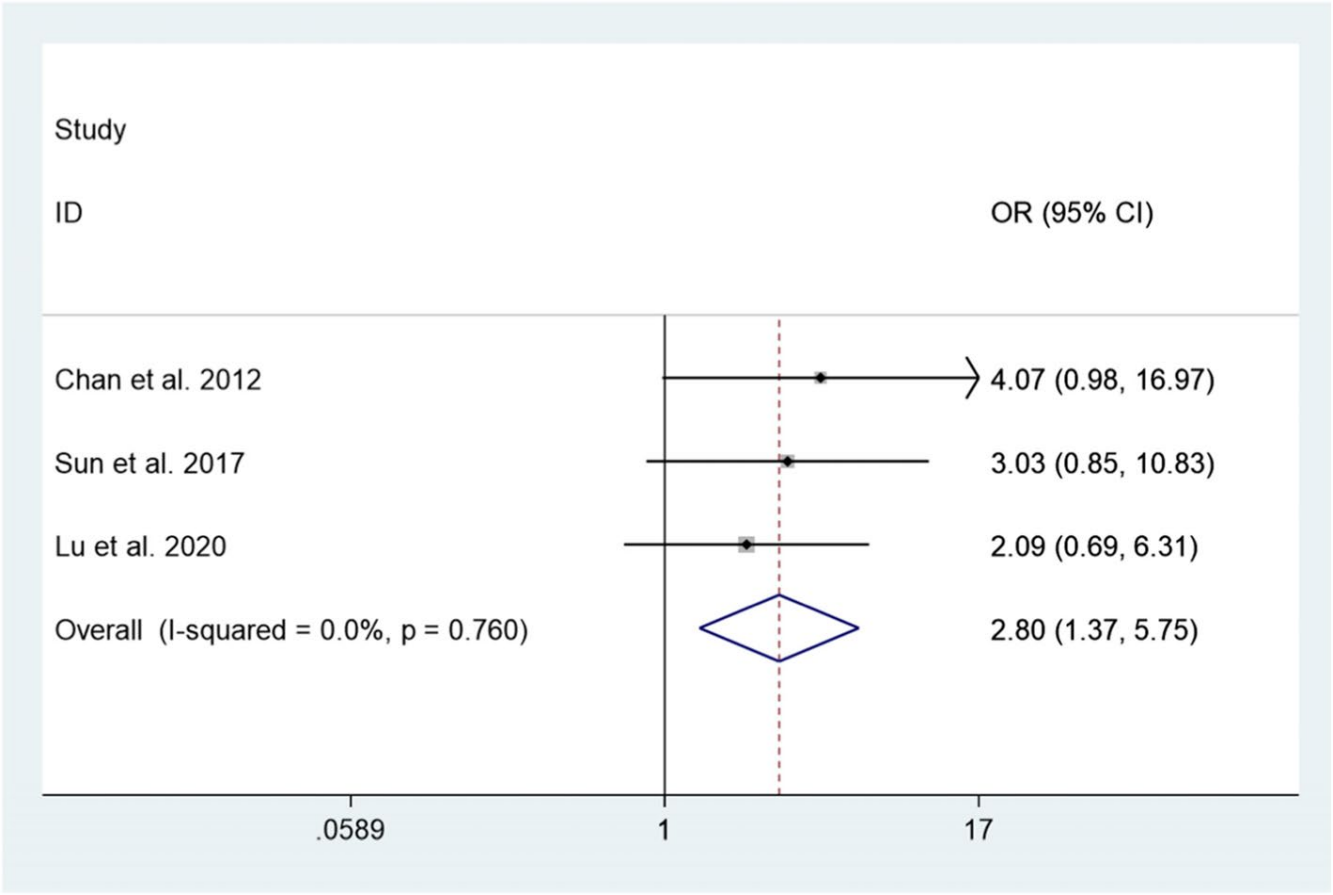
Comparison in the postoperative CD grade II or higher complication rate between repeated hepatectomy and radiofrequency ablation in the treatment of recurrent liver cancer

In addition, we explored the difference in the postoperative CD grade II or higher complication rate between RH and RFA. There are 3 studies [15, 20, 22], including 392 patients, compared the postoperative CD grade II or higher complication rate between RH and RFA. Since there is no significant heterogeneity (*I*^2^ = 0.0%, *P* = 0.760 > 0.1), a meta-analysis was conducted through a fixed effects model. The pooled results show that the postoperative CD grade II or higher complication rate of RH was significantly higher than RFA (*OR* = 2.80, 95% *CI*: 1.37–5.75, *P* = 0.005) (Fig. 4).

### Sensitivity analysis

Sensitivity analysis eliminates each included study one by one and performs a summary analysis on the remaining studies to assess whether a single included study has an excessive impact on the results of the entire meta-analysis. The result of the sensitivity analysis is shown in Additional file 1 (Fig. S1–7). The results showed that none of the studies had an excessive impact on the results of the meta-analysis, indicating that the results of the remaining studies are stable and reliable.

### Publication bias

**Fig. 5:**
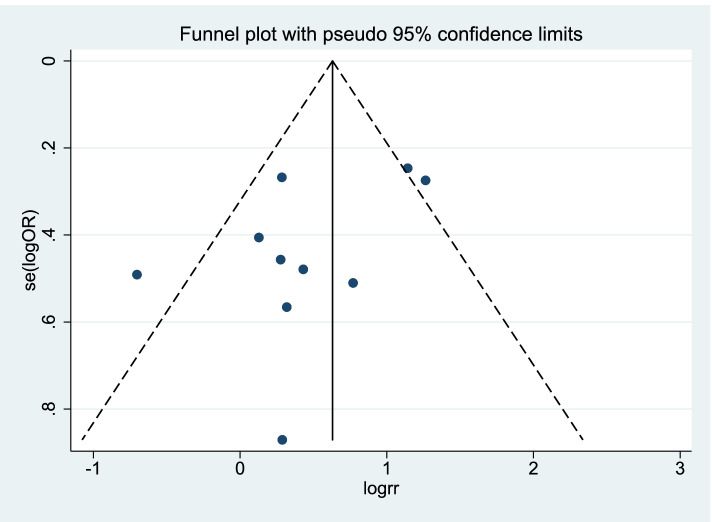
Funnel plot for evaluating the publication bias of this meta-analysis

The funnel plot of this study is shown in Fig. 5. It can be seen that the funnel plot is basically symmetrical, and the *P*-value of Egger’s test is 0.108, indicating that there is no obvious publication bias in this study.

## Discussion

Repeated hepatectomy is the preferred treatment for patients with intrahepatic recurrence of primary liver cancer and good liver function [23]. Yoshioka et al. [6] pointed out that although various methods have been used to treat recurrent liver cancer, hepatectomy is the only possible cure. The study of Chua et al. [24] showed that the cumulative 5-year survival rate of patients after repeated hepatectomy can reach 60%. Studies have shown that postoperative recurrence of liver cancer includes intrahepatic metastasis and multicenter occurrence [25], while intrahepatic metastasis is more likely to spread to the branch of the portal vein, causing tumor emboli to flow to adjacent branches of the same liver segment. RH is a routine option for recurrent HCC, but its indications are limited by insufficient residual liver volume and severe abdominal adhesions. However, RFA has gradually become an effective alternative due to its advantages of being minimally invasive, safe, and reproducible [17]. RH can provide a better opportunity to eradicate intrahepatic micrometastasis caused by the primary tumor, and RFA is inferior to RH in terms of local tumor control [26, 27]. Due to impaired liver function, postoperative tissue adhesion, and the impact of previous surgery on the anatomy, RH is more challenging than initial resection. However, Zhou et al. [6] compared the perioperative results after initial and repeated hepatectomy and found no significant statistical difference. With improvements in liver function assessment, surgical techniques, and perioperative care, more patients are likely to undergo surgical resection. This meta-analysis pooled 8 studies evolving 1332 patients to compare the efficacy of RH and RFA in the treatment of recurrent liver cancer.

Our pooled results show that the 3-year and 5-year OS rate of the repeated hepatectomy group was significantly higher than that of the radiofrequency ablation group, while there is no significant difference in the 1-year OS rate of repeated hepatectomy group and radiofrequency ablation group. Similarly, our pooled results show that the 3-year and 5-year DFS rate of the repeated hepatectomy group was significantly higher than that of the radiofrequency ablation group, while there is no significant difference in the 1-year DFS rate of repeated hepatectomy group and radiofrequency ablation group. This shows that repeated hepatectomy has a better long-term effect than radiofrequency ablation, which may be due to the high degree of selection of patients with good liver function and limited intrahepatic tumor spread. However, ablation techniques are improved by introducing the microwave ablation and treatment planning software for reasonable margin ablation and reaching A0 [26]. It is worth noting that repeated hepatectomy is only an available treatment option for some patients, and the repeated resection rate is 20% [28]. Our pooled results of postoperative complications show the postoperative CD grade II or higher complication rate of the repeated hepatectomy group was significantly higher than that of the radiofrequency ablation group, which means that radiofrequency ablation has a lower incidence of postoperative complications. However, only 3 studies reported complications rate, making the reliability of the results challenged. Recent studies have shown that adjuvant sorafenib therapy after resection in patients with hepatocellular carcinoma could prolong overall survival and recurrence-free survival and reduce recurrence rates without intolerable side effects, suggesting that adjuvant sorafenib may help improve efficacy and safety of repeat hepatectomy [29]. Furthermore, Lee et al. proposed that if liver resection is possible after neoadjuvant hepatic arterial infusion chemotherapy (HAIC), liver resection would provide better outcomes than HAIC alone. This suggests that in addition to sorafenib, neoadjuvant HAIC may also contribute to improving the efficacy and safety of repeat hepatectomy [30]. Since the primary goal of HCC patients is to improve the survival rate, RH is more suitable for the treatment of recurrent HCC, although the complication rate of RH is higher than that of RFA. Interestingly, recent studies have shown that associating liver partition and portal vein ligation for staged hepatectomy and two-stage hepatectomy with inter-stage portal vein embolization shows excellent technical feasibility and comparable long-term oncologic outcome in colorectal liver metastases, and future studies are necessary to explore the difference in efficacy between LH and these two technologies in metastatic liver cancer [31].

In addition to active treatment of recurrent liver cancer, the prevention and early diagnosis of liver cancer are also necessary. Hepatitis B virus (HBV) infection is an important factor in the occurrence of liver cancer. Studies have shown that the p53 signaling pathway may be a potential biomarker and therapeutic target for HBV-related HCC [32], which helps us to diagnose and prevent the occurrence and recurrence of liver cancer early, thereby reducing surgery rates and healthcare costs.

This meta-analysis also has the following limitations: first, most of the included literature is retrospective research, which is of low quality compared with randomized controlled trial research; some literatures published too long may lead to bias. Second, most of the studies are cohort studies, which may lead to a reduction in the quality of the study, and more large randomized controlled trials are needed to compare the efficacy and safety of RH and RFA in the future. Third, many patients who are not suitable for surgery were referred for RF ablation, and this will be a confounding factor. However, the confounding factors were not described in detail in the eight included studies; subgroup analysis could not be performed in this paper. Fourth, we has not conducted a manual search strategy additionally as complementary retrieval from key journals and conference proceedings, which could lead to the omission of eligible studies that were presented only with abstract. Fifth, only 3 studies reported complications rate, making the reliability of the results challenging. In the future, it is necessary to continue to update new studies on adverse reactions to further explore the differences in the incidence of adverse reactions between RH and RFA.

## Conclusion

Based on the pooled results of 8 existing retrospective studies, RH has a higher OS rate and DFS rate in the treatment of recurrent liver cancer, while the postoperative complication rate of RFA is lower. When survival is the primary goal, RH should be the first choice for recurrent liver cancer.

## Supplementary Information

**Additional file 1: Figure S1.** Sensitivity analysis of the comparison of the 1-year OS rate between repeated hepatectomy group and radiofrequency ablation group. **Figure S2.** Sensitivity analysis of the comparison of the 3-year OS rate between repeated hepatectomy group and radiofrequency ablation group. **Figure S3.** Sensitivity analysis of the comparison of the 5-year OS rate between repeated hepatectomy group and radiofrequency ablation group. **Figure S4.** Sensitivity analysis of the comparison of the 1-year DFS rate between repeated hepatectomy group and radiofrequency ablation group. **Figure S5.** Sensitivity analysis of the comparison of the 3-year DFS rate between repeated hepatectomy group and radiofrequency ablation group. **Figure S6.** Sensitivity analysis of the comparison of the 5-year DFS rate between repeated hepatectomy group and radiofrequency ablation group. **Figure S7.** Sensitivity analysis of the comparison of the postoperative CD grade II or higher complication rate between repeated hepatectomy group and radiofrequency ablation group.

**Additional file 2.** Pubmed, Embase, and Cochrane.

## Data Availability

The datasets are available from the corresponding author on reasonable request.

## Abbreviations

CD: Clavien-Dindo
DFS: Disease-free survival
NOS: Newcastle-Ottawa Scale
OS: Overall survival
RH: Repeated hepatectomy
RFA: Radiofrequency ablation
OR: Odds ratio
CI: Confidence interval
HAIC: Hepatic arterial infusion chemotherapy
HBV: Hepatitis B virus
