# Supplementary material for: Comparison of the efficacy and safety of repeated hepatectomy and radiofrequency ablation in the treatment of primary recurrent liver cancer: a meta-analysis
Source: World J Surg Oncol. 2022 Jun 6;20:182. doi: 10.1186/s12957-022-02649-4 (PMC9169306; doi:10.1186/s12957-022-02649-4)
Supplement: Supplementary file 1 — Additional file 1: Figure S1. Sensitivity analysis of the comparison of the 1-year OS rate between repeated hepatectomy group and radiofrequency ablation group. Figure S2. Sensitivity analysis of the comparison of the 3-year OS rate between repeated hepatectomy group and radiofrequency ablation group. Figure S3. Sensitivity analysis of the comparison of the 5-year OS rate between repeated hepatectomy group and radiofrequency ablation group. Figure S4. Sensitivity analysis of the comparison of the 1-year DFS rate between repeated hepatectomy group and radiofrequency ablation group. Figure S5. Sensitivity analysis of the comparison of the 3-year DFS rate between repeated hepatectomy group and radiofrequency ablation group. Figure S6. Sensitivity analysis of the comparison of the 5-year DFS rate between repeated hepatectomy group and radiofrequency ablation group. Figure S7. Sensitivity analysis of the comparison of the postoperative CD grade II or higher complication rate between repeated hepatectomy group and radiofrequency ablation group. [file 12957_2022_2649_MOESM1_ESM.docx]

**Sensitivity analysis**

Figure S1. Sensitivity analysis of the comparison of the 1-year OS rate between repeated hepatectomy group and radiofrequency ablation group.

Figure S2. Sensitivity analysis of the comparison of the 3-year OS rate between repeated hepatectomy group and radiofrequency ablation group.

Figure S3. Sensitivity analysis of the comparison of the 5-year OS rate between repeated hepatectomy group and radiofrequency ablation group.

Figure S4. Sensitivity analysis of the comparison of the 1-year DFS rate between repeated hepatectomy group and radiofrequency ablation group.

Figure S5. Sensitivity analysis of the comparison of the 3-year DFS rate between repeated hepatectomy group and radiofrequency ablation group.

Figure S6. Sensitivity analysis of the comparison of the 5-year DFS rate between repeated hepatectomy group and radiofrequency ablation group.

Figure S7. Sensitivity analysis of the comparison of the postoperative CD grade II or higher complication rate between repeated hepatectomy group and radiofrequency ablation group.
