# Supplementary material for: Comparison of the efficacy and safety of repeated hepatectomy and radiofrequency ablation in the treatment of primary recurrent liver cancer: a meta-analysis
Source: World J Surg Oncol. 2022 Jun 6;20:182. doi: 10.1186/s12957-022-02649-4 (PMC9169306; doi:10.1186/s12957-022-02649-4)
Supplement: Supplementary file 2 — Additional file 2. Pubmed, Embase, and Cochrane. [file 12957_2022_2649_MOESM2_ESM.docx]

Pubmed:

((((((((((((((((((((((("Liver Neoplasms"[Mesh]) OR (Neoplasms, Liver[Title/Abstract])) OR (Liver Neoplasm[Title/Abstract])) OR (Neoplasm, Liver[Title/Abstract])) OR (Hepatic Neoplasms[Title/Abstract])) OR (Hepatic Neoplasm[Title/Abstract])) OR (Neoplasm, Hepatic[Title/Abstract])) OR (Cancer of Liver[Title/Abstract])) OR (Hepatocellular Cancer[Title/Abstract])) OR (Cancers, Hepatocellular[Title/Abstract])) OR (Hepatocellular Cancers[Title/Abstract])) OR (Hepatic Cancer[Title/Abstract])) OR (Cancer, Hepatic[Title/Abstract])) OR (Cancers, Hepatic[Title/Abstract])) OR (Hepatic Cancers[Title/Abstract])) OR (Liver Cancer[Title/Abstract])) OR (Cancer, Liver[Title/Abstract])) OR (Cancers, Liver[Title/Abstract])) OR (Liver Cancers[Title/Abstract])) OR (Cancer of the Liver[Title/Abstract])) OR (Cancer, Hepatocellular[Title/Abstract])) AND ((((Recurrent[Title/Abstract]) OR (recurrence[Title/Abstract])) OR (relapse[Title/Abstract])) OR (recurring[Title/Abstract]))) AND (((((("Radiofrequency Ablation"[Mesh]) OR (Ablation, Radiofrequency[Title/Abstract])) OR (Radio Frequency Ablation[Title/Abstract])) OR (Ablation, Radio Frequency[Title/Abstract])) OR (Radio-Frequency Ablation[Title/Abstract])) OR (Ablation, Radio-Frequency[Title/Abstract]))) AND ((((((((((repeated hepatic resection[Title/Abstract]) OR (re-hepatectomy[Title/Abstract])) OR (repeat hepatectomy[Title/Abstract])) OR (hepatic resection[Title/Abstract])) OR (repeated resection[Title/Abstract])) OR (re-resection[Title/Abstract])) OR (liver resection[Title/Abstract])) OR (surgical resection[Title/Abstract])) OR (redo hepatectomy[Title/Abstract])) OR (repeat Liver Resection[Title/Abstract]))

Embase:

#1: 'liver neoplasms':ti,ab,kw OR 'liver neoplasm':ti,ab,kw OR 'hepatic neoplasms':ti,ab,kw OR 'hepatic neoplasm':ti,ab,kw OR 'cancer of liver':ti,ab,kw OR 'hepatocellular cancer':ti,ab,kw OR 'hepatocellular cancers':ti,ab,kw OR 'hepatic cancer':ti,ab,kw OR 'hepatic cancers':ti,ab,kw OR 'liver cancer':ti,ab,kw OR 'liver cancers':ti,ab,kw OR 'cancer of the liver':ti,ab,kw

#2: recurrent:ti,ab,kw OR recurrence:ti,ab,kw OR relapse:ti,ab,kw OR recurring:ti,ab,kw

#3: 'radiofrequency ablation':ti,ab,kw OR 'radio frequency ablation':ti,ab,kw OR 'radio-frequency ablation':ti,ab,kw

#4: 'repeated hepatic resection':ti,ab,kw OR 're hepatectomy':ti,ab,kw OR 'repeat hepatectomy':ti,ab,kw OR 'hepatic resection':ti,ab,kw OR 'repeated resection':ti,ab,kw OR 're resection':ti,ab,kw OR 'liver resection':ti,ab,kw OR 'surgical resection':ti,ab,kw OR 'redo hepatectomy':ti,ab,kw OR 'repeat liver resection':ti,ab,kw

#5: #1 AND #2 AND #3 AND #4

Cochrane:

#1：(liver neoplasms):ti,ab,kw OR (liver neoplasm):ti,ab,kw OR (hepatic neoplasms):ti,ab,kw OR (hepatic neoplasm):ti,ab,kw OR (cancer of liver):ti,ab,kw OR (hepatocellular cancer):ti,ab,kw OR (hepatocellular cancers):ti,ab,kw OR (hepatic cancer):ti,ab,kw OR (hepatic cancers):ti,ab,kw OR (liver cancer):ti,ab,kw OR (liver cancers):ti,ab,kw OR (cancer of the liver):ti,ab,kw

#2：(recurrent):ti,ab,kw OR (recurrence):ti,ab,kw OR (relapse):ti,ab,kw OR (recurring):ti,ab,kw

#3：(radiofrequency ablation):ti,ab,kw OR (radio frequency ablation):ti,ab,kw OR (radio-frequency ablation):ti,ab,kw

#4：(repeated hepatic resection):ti,ab,kw OR (re-hepatectomy):ti,ab,kw OR (repeat hepatectomy):ti,ab,kw OR (hepatic resection):ti,ab,kw OR (repeated resection):ti,ab,kw OR (re-resection):ti,ab,kw OR (liver resection):ti,ab,kw OR (surgical resection):ti,ab,kw OR (redo hepatectomy):ti,ab,kw OR (repeat Liver Resection):ti,ab,kw

#5: #1 AND #2 AND #3 AND #4
